# ADAM10-mediated release of heregulin confers resistance to trastuzumab by activating HER3

**DOI:** 10.18632/oncotarget.7200

**Published:** 2016-02-05

**Authors:** Eva A. Ebbing, Jan Paul Medema, Helene Damhofer, Sybren L. Meijer, Kausilia K. Krishnadath, Mark I. van Berge Henegouwen, Maarten F. Bijlsma, Hanneke W. M. van Laarhoven

**Affiliations:** ^1^ Laboratory for Experimental Oncology and Radiobiology, Center for Experimental and Molecular Medicine, Academic Medical Center, 1105 AZ Amsterdam, The Netherlands; ^2^ Department of Pathology, Academic Medical Center, 1105 AZ Amsterdam, The Netherlands; ^3^ Department of Gastroenterology and Hepatology, Academic Medical Center, 1105 AZ Amsterdam, The Netherlands; ^4^ Department of Surgery, Academic Medical Center, 1105 AZ Amsterdam, The Netherlands; ^5^ Department of Medical Oncology, Academic Medical Center, 1105 AZ Amsterdam, The Netherlands; ^6^ Cancer Genomics Center, Academic Medical Center, 1105 AZ Amsterdam, The Netherlands

**Keywords:** ADAM10, HER3, trastuzumab, NRG-1β, esophageal cancer

## Abstract

Receptor tyrosine kinases of the HER-family are involved in the development and progression of multiple epithelial tumors, and have consequently become widely used targets for new anti-cancer therapies. Trastuzumab, an antibody against HER2, has shown potent growth inhibitory effects on HER2 overexpressing tumors, including gastro-esophageal cancer, however, resistance to this therapy is inevitable. Unfortunately, a paucity of data on the cellular mechanisms of resistance to targeted therapeutic agents exists in esophageal adenocarcinoma. Using primary established HER2-overexpressing cultures and patient-derived xenograft models, we now reveal a novel resistance mechanism to trastuzumab in esophageal cancer: In response to trastuzumab, both HER3 and the metalloprotease ADAM10 are simultaneously upregulated. The proteolytic activity of the latter then releases the HER3 ligand heregulin from the cell surface to activate HER3 and confer resistance to trastuzumab by inducing compensatory growth factor receptor signaling. Blocking either HER3 or ADAM10 effectively reverts the acquired resistance to trastuzumab. Our data thus provide strategies to inhibit this signaling and circumvent resistance to trastuzumab.

## INTRODUCTION

Receptor tyrosine kinases (RTKs) of the human epidermal growth factor receptor (HER) family are involved in the development and progression of multiple epithelial tumors including esophageal adenocarcinoma (EAC) [1–4]. Dimerization of and between these receptors results in the phosphorylation of their intracellular domains, stimulating downstream signaling pathways that activate for instance cell proliferation, and enhanced cell motility [5]. As a consequence, HER family members have been regarded as prime candidates for anti-cancer targeting therapies using small molecule inhibitors or humanized monoclonal antibodies [5–7].

Members of the HER family, including HER2, can form hetero- or homodimers. Although no activating ligand is known for this receptor, the most potent signaling by HER2 is mediated by the heterodimers it forms with HER3. Activation of this dimer is initiated by binding of the ligand for HER3, heregulin (neuregulin-1β, NRG-1β) [8, 9]. Signaling of the neuregulin ligands through their matching HERs mediates important processes in developmental biology, the adult organism, but also in the development and progression of disease [10]. NRG-1β is a product of one of the sixteen different transcripts of neuregulin-1, and induces therapy resistance to the EGFR-targeting antibody cetuximab in colorectal cancer, RAF inhibitors in BRAF mutant melanoma, and MEK inhibitors in metastatic uveal melanoma [11–15]. The activation of NRG-1β requires proteolytic cleavage, as it is produced as a transmembrane protein. Proteases that can cleave the different neuregulin variants include the disintegrin and metalloproteinase proteins ADAM10 and ADAM17 [16, 17].

Whether a given esophageal tumor overexpresses HER2 seems to depend on the location in the organ and histological subtype. Overexpression has been described to be between 0-43%. The adenocarcinoma subtype is most often HER2 positive (10-43%) and is therefore a likely candidate for HER2-inhibitory treatment [2, 18]. Indeed, the phase III ToGA trial showed a significant survival benefit of trastuzumab, a humanized antibody against HER2, combined with chemotherapy compared to chemotherapy alone in patients with advanced-stage esophageal or gastric adenocarcinomas [19]. However, the improvement in survival was modest and, importantly, even patients with an initial response to trastuzumab eventually showed progression. Thus, resistance to trastuzumab is a major problem in esophagogastric cancer patients [20, 21].

The mechanisms of resistance against trastuzumab are various and include the upregulation of other tyrosine kinase receptors such as IGF1R or MET receptor [22–24], or reactivating mutations and protein overexpression in the pathway downstream of HER2, including PTEN, PI3K, and c-SRC. These have mainly been defined in breast cancer [25–27]. In EAC, however, mutations in HER2 or its downstream signaling pathway components are not common and the mechanisms of resistance in this disease are unclear [28]. Given the high degree of specificity of trastuzumab for HER2, upregulation and activation of HER family members other than the targeted receptor could occur. Of these, HER3 is the most likely candidate because of its involvement in resistance mechanisms in other cancer types, including trastuzumab-induced resistance in breast cancer and resistance to MEK inhibition in KRAS-mutant lung and colon cancer [29, 30]. Unfortunately, a paucity of data on the expression of HERs in EAC other than EGFR and HER2 exists, and so far limited data on HER3 levels and expression dynamics in EAC have been reported [31, 32]. In this study, we defined the dynamics of HER family member expression during HER2-targeting treatment and in doing so identified a entirely novel mechanism of resistance. Resistance is induced by the concomitant upregulation of both HER3 and the metalloprotease ADAM10. The proteolytic activity of the latter protein then leads to the release of the HER3 ligand NRG-1β, which in its turn is able to re-activate the HER2/HER3 signaling axis. This compensatory signaling can be blocked at several levels, namely by the inhibition of HER3 or ADAM10, resulting in an abrogation of resistance to trastuzumab.

## RESULTS

### Long term trastuzumab treatment is accompanied by the upregulation of EGFR and HER3

**Figure 1:**
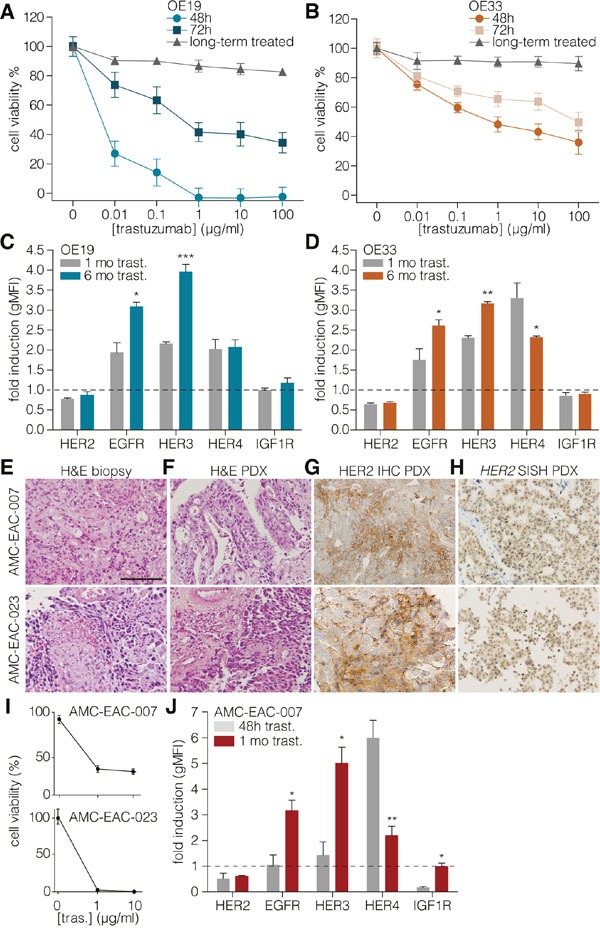
Long-term HER2 targeting causes upregulation of EGFR and HER3 **A.** OE19 and OE33 **B.** cells were treated with indicated concentrations of trastuzumab for 48 or 72h in fully supplemented medium. The long-term condition was first cultured for one month in trastuzumab supplemented medium before used in this assay. In this condition, cells were plated without treatment prior to 48h trastuzumab treatment with the indicated concentrations. Cell viability was measured using the Cell Titer Blue assay and values were normalized to untreated cells. Data show the mean ± s.e.m., of three independent experiments. **C.** OE19 and OE33 **D.** cells were cultured for 1 month (gray bars) or 6 months (colored bars) in medium containing 1μg/ml trastuzumab. Cell surface expression of HERs was determined by flow cytometry. Values represent the gMFI ± s.e.m and are normalized to short-term (48 hours) trastuzumab treatment, n≥3. (* = *P*-value <0.05, ** = *P*-value <0.01, *** = *P*-value <0.001). **E.** Representative H&E images of AMC-007-EAC (upper row) and AMC-023-EAC (lower row) patient biopsies from the primary tumor. Scale bar: 100μm; **F.** H&E of the derived PDX; **G.** immunohistochemistry for HER2 on PDX; **H.** Silver in situ hybridization (SISH) for *HER2* on PDX. **I.** Cell viability following 48 h 1μg/ml trastuzumab treatment was determined by Cell Titer Blue and values were corrected for input and normalized to untreated cells, n≥3. **J.** AMC-EAC-007 cells cultured for 48 h (gray bars) or 1 month (colored bars) in 1μg/ml trastuzumab containing medium. Cell surface expression of receptors was determined using flow cytometry. Values represent the geometric MFI ± s.e.m and are normalized to untreated cells, n≥3. (* = *P*-value <0.05, ** = *P*-value <0.01).

In order to assess the mechanisms of resistance to trastuzumab in EAC, we measured the sensitivity to this drug over time in two HER2-positive EAC cell lines; OE19 (highly overexpressing HER2) and OE33 (intermediate HER2 overexpression, see also Supplementary Figure S1). A dose-dependent effect of trastuzumab on cell viability was observed, and HER2 expression levels correlated with the magnitude of response (Figures 1A and 1B). Surprisingly, both cell lines showed a reduced sensitivity to trastuzumab within a relatively short time frame, seemingly incompatible with a selection for clones harboring mutated signaling components. Instead, a possible explanation for this resistance could be the upregulation of compensatory receptors other than HER2 to activate shared downstream signaling components. To determine which HER family members might activate such signaling, EAC cells were treated with trastuzumab, and surface levels of candidate receptors were determined.

Of the receptors analyzed following short-term (48h) treatment, HER4 showed a marked upregulation as determined by flow cytometry and transcript analysis (Supplementary Figures 2A–E). Inducibly HER4-silenced OE19 cells showed a restored sensitivity to trastuzumab up until 72 hours, which was again lost after 96 hours (Supplementary Figure 2F). We concluded from this that HER4 is likely not sufficient to drive resistance over the longer periods that are relevant to human disease. We hypothesized other RTKs to be responsible for this instead.

In order to determine which HER family members could induce long-term resistance to trastuzumab, cells were cultured continuously with trastuzumab from 1 up till 6 months, and cell surface levels of HERs were measured (Figures 1C and 1D). HER2 levels were still decreased. Surface HER4 levels were lower compared to short-term treated cultures. Instead, EGFR and HER3 expression levels were now increased, suggesting a role for these RTKs in the resistance against trastuzumab over extended periods of time. To assess if the mechanism of resistance to trastuzumab observed in the cell lines also held true in primary material, we established patient derived xenografts from HER2-overexpressing tumors and derived cultures from these (Figures 1E – 1H) [33]. These cells were sensitive to trastuzumab (Figure 1I), and were subsequently cultured with trastuzumab for extended periods of time. HER dynamics were measured by flow cytometry and revealed the same pattern of EGFR and HER3 upregulation as the cell lines (Figure 1J).

**Figure 2:**
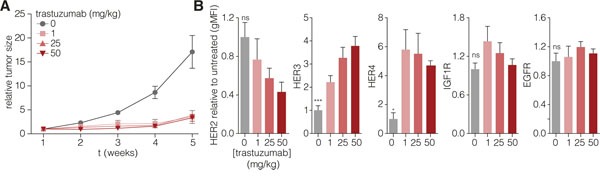
HER2 targeting *in vivo* induces resistance and concomitant upregulation of HER3 **A.** Tumor pieces derived from AMC-EAC-007 passage two were processed to yield equally sized pieces of 2 mm^3^ and subcutaneously grafted with Matrigel (BD) into the flank of NSG mice. Mice with tumors reaching a size of 100 mm^3^ were injected intraperitoneally with 1, 25, or 50 mg/kg trastuzumab, once a week, for the duration of 4 weeks (5 mice per group). Tumor growth was measured every week prior to trastuzumab injection. Values are normalized to tumor size at the start of treatment. **B.** A week after the last (4^th^) injection, tumors were harvested and surface levels were assessed for the indicated receptors. Values represent the mean gMFI ± s.e.m., and are normalized to the untreated control group, one-way ANOVA was used to determine statistical significance which is indicated on the grey bars, ns (not significant). (* = *P*-value <0.05, ** = *P*-value <0.01).

To assess whether the resistance to trastuzumab by upregulation of compensatory receptors is conserved *in vivo*, we treated tumor-bearing mice (AMC-EAC-007) with trastuzumab and assessed tumor growth, and HER levels after the experiment. As expected for a HER2-positive tumor, a strong inhibitory effect of trastuzumab on tumor growth was observed for all tested doses (Figure 2A). However, tumor regrowth was observed after 4 weeks despite treatment, and we concluded that by this time the tumors had developed resistance. When HER levels were assessed (Figure 2B), a clear dose-dependent decrease in HER2 was observed, as well as a significant increase of HER3 and HER4. IGF1R levels remained constant, and in contrast to the *in vitro* data, no increase in EGFR levels was observed either. Thus, the upregulation of HER3 is the most conserved and consistent response following HER2 inhibition.

### Targeting HER3 overcomes resistance to trastuzumab

**Figure 3:**
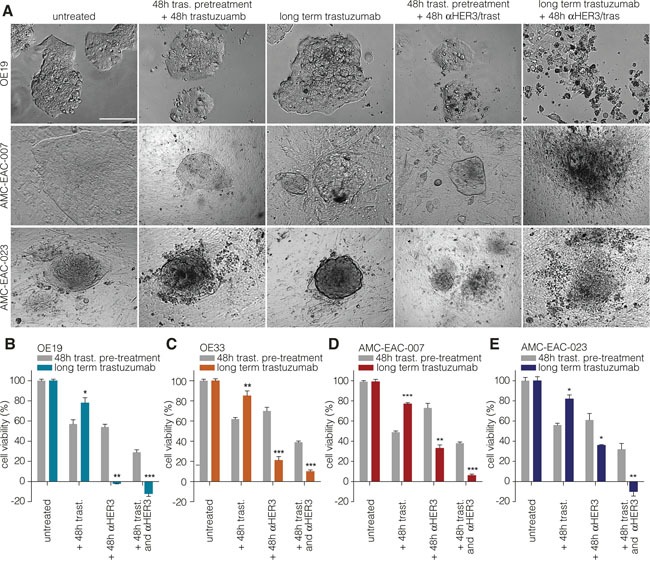
Targeting HER3 overcomes resistance to trastuzumab in cell lines and primary cells **A.** Representative images of OE19 (upper row), AMC-007-EAC (middle row), AMC-023-EAC (lower row) treated with the indicated compounds. Trastuzumab was used at 1μg/ml; a-HER3 at 0.1μg/ml. Scale bar: 100μm. **B – E.** The short term treated cells were first treated with trastuzumab for 48h, followed by a 48h incubation with either a-HER3 alone or a combination of trastuzumab and a-HER3. As a control, cells were first cultured short-tern (48h) with trastuzumab followed by an additional trastuzumab treatment period of 48h. Trastuzumab indicated on x-axis means that treatment was continued during the experiment along with the other indicated treatments. Cell viability was measured using CTB assay prior to treatment (input) and after treatment. Values are corrected for input and normalized to the control treated condition. Data show the mean ± s.e.m., n≥3. (* = *P*-value <0.05, ** = *P*-value <0.01, *** = *P*-value <0.001).

To assess the functional relevance of the observed upregulation of HER3, blocking antibody (a-HER3) was added to long-term and short-term trastuzumab treated cells and cell morphology was assessed (Figure 3A). Long-term treatment with trastuzumab in combination with a-HER3 resulted in a reduction of cell viability and induction of cell death in two cell lines and two primary cultures established from patient-derived xenografts (Figure 3A – 3E). Furthermore, cell viability assays showed increased sensitivity towards panitumumab (a humanized antibody directed against EGFR) in the long-term trastuzumab treated cells (Supplementary Figure S3). However, HER3 inhibition (Figure 3A) was the most effective treatment compared to EGFR inhibition (Supplementary Figure 3). Taken together, these data show a consistent mechanism of resistance upon long-term trastuzumab treatment through upregulation of HER3, and show that inhibition of this receptor can circumvent resistance to trastuzumab.

### Neuregulin-1β induced HER3 activation is mediated by ADAM10

**Figure 4:**
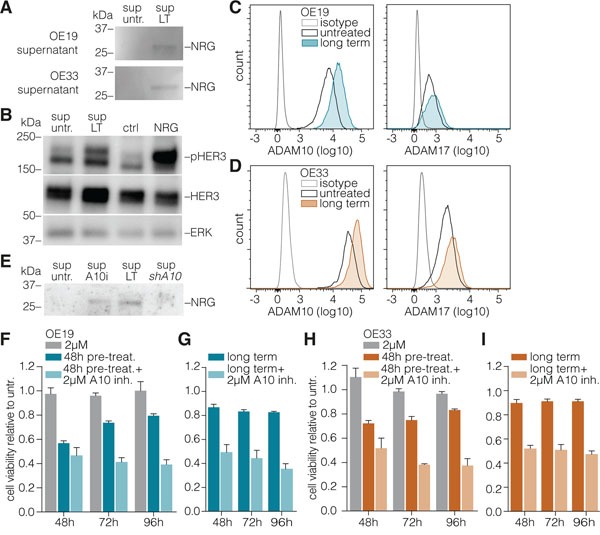
ADAM10 mediates neuregulin-1β release to activate HER3 **A.** Medium was incubated on equal numbers of long-term treated or control OE19 cells for 96h. Supernatants were harvested, cells and debris were cleared from the supernatant by centrifugation, and processed for Western blotting against NRG-1β without concentrating supernatant proteins. **B.** CC09 primary colon cancer stem cells were treated for 10min with supernatants from experiments as shown in panel A, as well as recombinant NRG-1β (at 2 ng/mL) or control. Cells were processed for Western blotting, using antibodies against phosphorylated HER3 and total HER3. ERK1/2 was used as loading control. **C. D.** OE19 and OE33 cells were treated long-term with trastuzumab (or control) and surface levels of ADAM10 (left column) and ADAM17 (right column) were assessed by FACS. **E.** NRG-1β levels were measured in the supernatants of OE19 cells as for panel a, using an equal amount of control (untreated) cells, and cells cultured long-term with trastuzumab (sup LT), treated with 2μM ADAM10 inhibitor 72h prior to supernatant incubation (sup A10i), or stably transduced with a silencing hairpin against ADAM10 (sup shA10). **F.** OE19 cells were either untreated or pre-treated for indicated times with trastuzumab prior to the addition of ADAM10 inhibitor (2μM, 48 h) and cell viability was assessed. Plotted are Cell Titer Blue assay data relative to untreated (set to 1, not shown in graph); mean ± s.e.m., n=9. **G.** As for panel **F**, using long-term trastuzumab treated OE19 cells. **H. I.** As for panels **F** and **G**, using OE33 cells.

In contrast to HER2, HER3 requires ligand for its activation and its upregulation *per se* cannot account for activation of its downstream pathway. Therefore, we measured known ligands of HER3 in our experimental setup, and found NRG-1β in the supernatant of long-term trastuzumab treated cells. This ligand was absent from control conditions (Figure 4A). To determine if this NRG-1β was biologically active, we used a primary colon cancer line (CC09) that expresses HER3 but not the ligands for this receptor as a reporter [34]. Supernatant of long-term treated OE19s was indeed found to contain biologically active NRG-1β, inducing HER3 phosphorylation in CC09 cells (Figure 4B).

NRG-1β needs to be released from the cell surface for its dissemination and activity. This is typically induced by the enzymatic action of dedicated proteins like the ADAMs, and we hypothesized the release of HER3 ligand in the supernatant of the long-term trastuzumab treated cells to also be a product of proteolytic cleavage. Levels of the two best characterized metalloproteases involved in HER ligand shedding, ADAM10 and −17 [16] were determined following long-term trastuzumab treatment. Increased levels of ADAM10 were observed in response to trastuzumab (Figures 4C and 4D). To functionally assess if this ADAM10 is involved in the release of NRG-1β, cells were either treated with an ADAM10 inhibitor, or transduced with silencing RNA against ADAM10. Analysis of the supernatants of these cells indeed showed a decreased NRG-1β release by those cells of which ADAM10 function was inhibited (Figure 4E).

**Figure 5:**
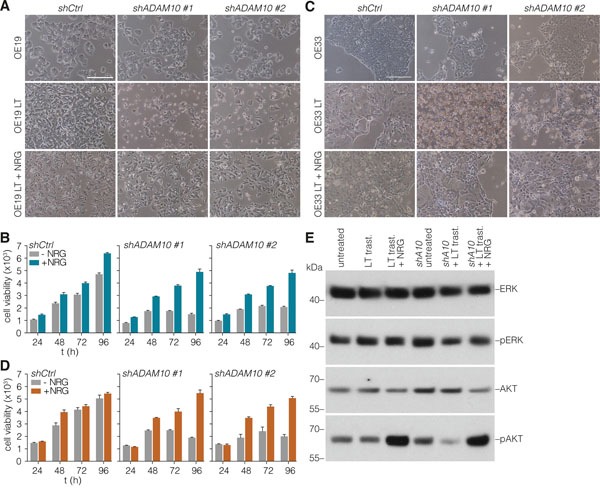
Loss of ADAM10 function can be rescued by addition of exogenous NRG-1β **A.** OE19 cells (control or long-term treated) were silenced for ADAM10 by stable shRNA transduction, and morphology was assessed by microscopy. Medium was supplemented with exogenous NRG-1β at 2 ng/ml. **B.** Cells were treated as for panel A, and cell viability was assessed using Cell Titer Blue. Plotted are data relative to unsupplemented (control treated) cells at 24h set to 1; shown is mean ± s.e.m., n=9. **C.** As for panel A, using OE33 cells. **D.** As for panel B, using OE33 cells. **E.** Western blot for ERK (Thr202/Tyr204) and AKT (Ser473) phosphorylation under the indicated conditions in OE19 cells: untreated, long-term trastuzumab treated (LT trast.), long-term trastuzumab treated + recombinant NRG-1β (2 ng/mL), untreated + ADAM10 knockdown (shA10), long-term trastuzumab treated + ADAM10 knockdown, long-term trastuzumab treated + ADAM10 knockdown + recombinant NRG-1β. Cells treated with recombinant NRG-1β were incubated for 10 minutes.

To address whether the ADAM10-induced release of NRG-1β is required for HER3-mediated resistance, untreated, short-term, or long-term trastuzumab treated cells were incubated with ADAM10 inhibitor and a reversal of resistance to trastuzumab was observed in the latter condition (Figures 4F – 4I). Similarly, no effect of ADAM10 knockdown was observed in otherwise untreated cells by microscopy (Figures 5A and 5C, upper row) and cell viability assays (Figures 5B and 5D, left panels), while in long-term trastuzumab treated cells, ADAM10 knockdown decreased cell numbers (Figures 5A and 5C, middle row). This effect could be rescued by the addition of exogenous NRG-1β ligand, as shown by microscopy (Figures 5A and 5C, lower row) and cell viability assays (Figures 5B and 5D). To assess the downstream signaling effects of long-term HER2 inhibition and ADAM10 knockdown, AKT and ERK phosphorylation was determined under these conditions. AKT phosphorylation remained stable following long-term trastuzumab treatment, while it decreased upon the addition of ADAM10 knockdown (shA10 + LT trast.). The addition of recombinant NRG-1β was able to restore AKT phosphorylation in all conditions, thus rescuing growth factor receptor signaling upon ADAM10 loss (Figure 5E). ERK phosphorylation remained constant throughout all experimental conditions.

## DISCUSSION

We have identified an intricate non-genetic sequence of events underlying resistance to trastuzumab in EAC. The concomitant upregulation of a compensatory receptor (HER3), as well as the metalloprotease (ADAM10) required for the release and activation of HER3 ligand (NRG-1β), function in union to induce resistance. Despite the apparent complexity of this mechanism, we found it to be remarkably consistent between the different experimental models tested. Although we also observed increased EGFR levels upon trastuzumab treatment *in vitro*, this effect was not observed *in vivo*. This difference could be explained by the presence of factors in the extra-cellular matrix, obviating the need for overexpression of EGFR in vivo. Furthermore, the randomized phase III trial TRIO-013/LOGiC showed no additional effect of lapatinib, a dual tyrosine kinase inhibitor of EGFR and HER2, on the overall survival of HER2 positive gastroesophageal adenocarcinoma patients treated with capecitabine and oxaliplatin [35]. Therefore, we take the observed *in vitro* increase of EGFR to be a less consistent and clinically relevant mechanism of trastuzumab-induced resistance than the upregulation of HER3.

Previous studies in other solid malignancies, including gastric cancer, have shown that overexpression of HER3 correlates with poor outcome [36]. Trastuzumab-induced resistance through various receptor tyrosine kinases including IGF1R and HER3 has been described in ovarian and breast cancer [37, 38], and the latter study also described antitumor activity of a combination of trastuzumab and a HER3 inhibitor on trastuzumab-resistant cells. Furthermore, phase-I dose-finding and feasibility studies are currently running for advanced solid tumors using humanized anti-HER3 monoclonal antibodies [39–41]. Although none of these studies have provided an explanation as to the source of HER3-activating ligand, they do highlight the urgency of HER3 activation in tumor progression and acquired resistance [42].

The interactions between the different HER family members can activate common downstream signaling pathways.[5] However, they rely on different activation mechanisms at the cell surface, which has consequences for the efficacy of HER inhibiting strategies. For instance, the favored and most potent dimers are HER2-3 heterodimers and although trastuzumab is an effective HER2 dimerization inhibitor, it cannot prevent dimerization with ligand-bound HER3 [43, 44]. Our finding that increased HER3 ligand release induces resistance to trastuzumab, can therefore also be explained by the fact that more of this ligand will be bound to the HER3 receptor and facilitate the formation of active HER2-3 heterodimers that are insensitive to trastuzumab. Blocking the HER3 receptor is therefore effective not only by blocking HER3 that is upregulated in response to the loss of HER2, but also by targeting the interaction between HER3 and HER2.

Of the ADAM family of metalloproteases, ADAM10 and ADAM17 are the best described members to shed HER ligands [16]. The release and activation of NRG-1β by ADAM10 and −17 has mainly been studied in the peripheral nervous system [45–47] and developmental biology [48]. However, two recent papers reported on the correlation of ADAM10 with survival in HER2-positive breast cancer [49], and NRG-1b-HER3-HER2 signaling was shown to promote trans-endothelial migration of breast cancer cell lines [50]. These considerations underscore the relevance of our finding that the release of HER-activating ligand in esophageal cancer is in fact induced by upregulated ADAM10.

Several clinically relevant consequences can be inferred from our work. One is that HER2 positive EAC is apparently eminently targetable with a low dose of trastuzumab. Furthermore, the consistent role for HER3 and the required release of its ligand by ADAM10 implies that in any HER2-positive EAC that develops resistance to HER2-inhibiting drugs, HER3, ADAM10 and NRG-1β can all be effective secondary targets. The first clinical trials directed at HER3 inhibition are currently running, and may prove to be a feasible strategy to safeguard HER2-targeting therapies from failure.

## MATERIALS AND METHODS

### Cell culture and chemicals

OE19 and OE33 (ATCC, Manassas, VA) were maintained in RPMI with 10% fetal bovine serum (FBS), L-glutamine (2mM), penicillin (100 units/mL), and streptomycin (500 μg/mL) (Lonza, Basel, Switzerland) according to routine cell culture procedures. HEK293T cells were maintained in high-glucose DMEM medium and supplemented as mentioned above. Long-term treated cells were continuously cultured with 1μg/ml trastuzumab for a duration of 1-6 months (Roche, Grenzach-Wyhlen, Germany). Trastuzumab, panitumumab (Amgen, London, England), and pertuzumab (Roche) were all kindly provided by the Academic Medical Center pharmacy. Anti-HER3 antibody (H3.105.5) was purchased from Millipore (Temecula, CA). ADAM10 inhibitor GI 254023X and recombinant hNRG-1β were purchased from R&D systems (Oxon, United Kingdom). NRG-1β was used at 2ng/ml.

### Constructs and lentiviral transduction

Hairpins against HER4 and ADAM10 from the Sigma Mission TRC library were cloned into pLKO-Tet-On or pLKO, respectively (primer sequences shown in Table S1). Clones were verified by sequencing. Virus production was performed as previously described [51]. To exclude an effect of doxycycline on the cells, control silenced cells were also doxycycline treated.

### Flow cytometry

Cells were harvested with trypsin-EDTA (Lonza) and washed in FACS buffer (PBS containing 1% FBS). 5×10^5^ cells were stained in a volume of 25μl for 30 minutes at 4°C. Antibodies were diluted in FACS buffer using the following concentrations: FITC conjugated a-HER2 affibody (1:1500, Bromma, Sweden); a-EGFR (1:2000, clone H11, DAKO, Carpinteria, CA); a-HER3 (1:1500, clone SGP1, Abcam); a-HER4 (1:200, clone H4.77.16, Abcam); a-IGFR (1:50, clone 33255, R&D, Minneapolis, MN); ADAM10 (1:500, MAB1427, R&D); ADAM17 (1:100, MAB9301, R&D). Secondary APC labeled a-mouse (550826, BD) was diluted 1:800. After washing, cells were resuspended in FACS buffer containing 50ng/ml propidium Iodide (PI) (Sigma) and acquired on a FACSCanto II (BD, Franklin Lakes, NJ). Data were analyzed with FlowJo 10 (Tree Star, Ashland, OR). The geometric mean fluorescence (gMFI) intensity in the relevant channel was calculated from the PI negative gate; gMFI from the isotype control was subtracted from the sample yielding the delta gMFI.

### Cell viability

Cell viability was determined using the Cell Titer-Blue Cell Viability Assay kit (Promega). Prior to the assay, cells were either cultured under normal conditions as described above or as for the long-term treated condition; cells were cultured in trastuzumab supplemented medium and were refreshed/passed twice a week. Cells were seeded into 96-well plates at a density of 5000 cells/well in triplicates in culture medium without trastuzumab. Of note, for the xenograft-derived cells, a confluent monolayer of fibroblasts from the same tumor was established prior to plating. 18 h after plating, baseline cell viability was measured, treatment was started (see below), and 20 μL of Cell Titer-Blue reagent was added to each well and incubated for 3 h. Plates were read in a cytofluormeter (BioTek Instruments, Winooski, VT). Percentage of cell growth inhibition was calculated by comparing the values obtained from treated versus control cells, minus the baseline cell viability or monolayer fibroblasts measured at 18 h after plating. Control groups were treated with PBS. For combination treatments, cells were either pre-treated for the indicated time in the graphs with 1μg/ml trastuzumab or the same volume of PBS in fully supplemented medium at normal culture conditions. This, followed by an additional 48 h treatment with either 0.1μg/ml panitumumab, a-HER3, or 2μM ADAM10 inhibitor alone or in combination with trastuzumab. In order to observe the effect of duration of trastuzumab treatment, the experiments were performed using trastuzumab-naïve cells, and cells that had been cultured continuously with 1μg/ml trastuzumab for 1-6 months. Pre-treatment with trastuzumab for 48 h was considered short-term, while treatment with trastuzumab during 1-6 months was considered long-term (LT).

### Patient derived xenografts and establishment of primary cell lines

Collection of material from patients diagnosed with esophageal adenocarcinoma in the Academic Medical Center (Amsterdam, The Netherlands) was approved by the institute's ethical committee (MEC 01/288#08.17.1042), and as described previously [33]. Informed consent was obtained from each patient. Animals for xenografts were bred and maintained at the local animal facility according to local legislation and ethical approval was obtained (LEX102774). Xenografts were processed as previously shown [33]. Animals were anesthesized with 1% isoflurane during grafting.

### *In vivo* experiment

Second passage xenografts were processed to yield equal sized pieces of 2 mm^3^ and grafted subcutaneously into the flank of NSG mice with Matrigel (BD). Mice with tumors reaching 100 mm^3^ were injected intraperitoneally with 1, 25, or 50mg/kg trastuzumab once a week for the duration of 4 weeks (n= 5 mice per dose). Tumor growth was measured weekly. Five weeks after start of treatment tumors were harvested and receptor expression was assessed by flow cytometry analysis. Animals in this experiment were bred and maintained at the local animal facility according to local legislation and ethical approval was obtained (LEX103096).

### Western blot

Cells were lysed in RIPA buffer (Cell Signaling, Beverly, MA) containing phosphatase and protease inhibitor cocktail (Cell Signaling). Protein levels were determined by BCA (Pierce). Samples were subjected to SDS-PAGE and transferred to PVDF membranes, blocked with 5% BSA (Lonza) in Tris-buffered saline with 0.1% Tween-20 (TBS-T), and incubated overnight at 4°C with primary antibodies; a-heregulin; a-ERK1/2 (Cell Signaling, clone 137F5, #4695) or a-pERK1/2, Thr202/Tyr204 (Cell Signaling, clone 197G2, #4377); a-HER3 (Cell Signaling, clone D22C5, #12708) or a-pHER3, Tyr1289 (Cell Signaling, clone D1B5, #2842); a-AKT (Cell Signaling, #9272) or a-pAKT, Ser473 (Cell Signaling, clone 193H12, #4058). All used 1:1.000. Goat anti rabbit horseradish peroxidase (HRP)-conjugated secondary antibodies was used at 1:10.000 (Cell Signaling, #7074). Proteins were either imaged using a FuijFilm LAS 4000 imager, using Lumi-Light plus western blot substrate (ROCHE, 12015196001) (Figure 4) or were develop on film using enhanced bioluminescence for HRP (ECL) was from GE Healthcare (Waukesha, WI, USA) (Figure 5).

### Quantitative PCR

Cells or tumor tissue was lysed using Trizol (Invitrogen) and RNA was isolated according to standard procedures. cDNA was synthesized using Superscript III (Invitrogen) in accordance with the manufacturer's protocol. Quantitative RT-PCR was performed using SYBR green (Roche, Basel, Switzerland) on a Lightcycler LC480 II (Roche). Relative expression values were calculated and normalized to a reference gene GAPDH according to the comparative threshold cycle (Cp) method. Primers were designed with Oligo Analyzer Version 3.1 Software. Primer sequences are listed in Table S1.

### Statistics

Differences between conditions indicated in the figures were assessed using Student's t*-*test or, in case of multiple groups, with one-way ANOVA. A P value of <0.05 was considered statistically significant. All values are represented as the mean ± s.e.m. derived from three independent biological replicates. Analyses were carried out using Graph Pad Prism 5 (GraphPad Software, La Jolla, CA or SPSS 10.1 software (SPSS, Inc., Chicago, IL).

## SUPPLEMENTARY FIGURES AND TABLE



## Abbreviations

ADAM: a disintegrin and metalloproteinase domain-containing protein
EAC: esophageal adenocarcinoma
HER: human epidermal growth factor receptor
RTK: receptor tyrosine kinase
