# Supplementary material for: ADAM10-mediated release of heregulin confers resistance to trastuzumab by activating HER3
Source: Oncotarget. 2016 Feb 5;7(9):10243–54. doi: 10.18632/oncotarget.7200 (PMC4891117; doi:10.18632/oncotarget.7200)
Supplement: Supplementary file 1 [file oncotarget-07-10243-s001.pdf]

SUPPLEMENTARY FIGURES AND TABLE

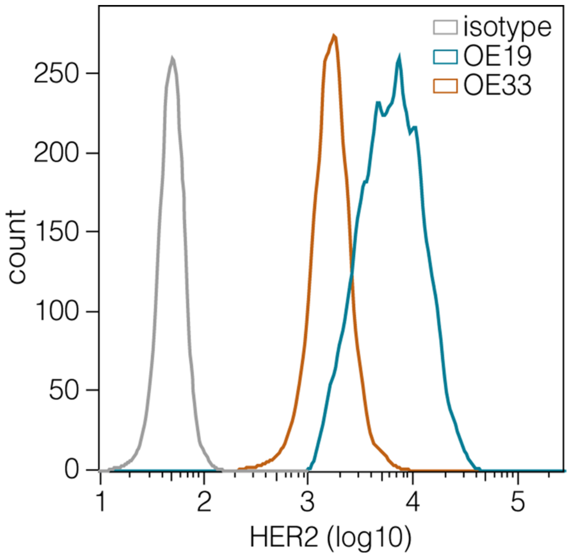

Supplementary Figure S1: HER2 cell surface expression. Representative flow cytometry staining for HER2 on EAC cell line OE19 and OE33.

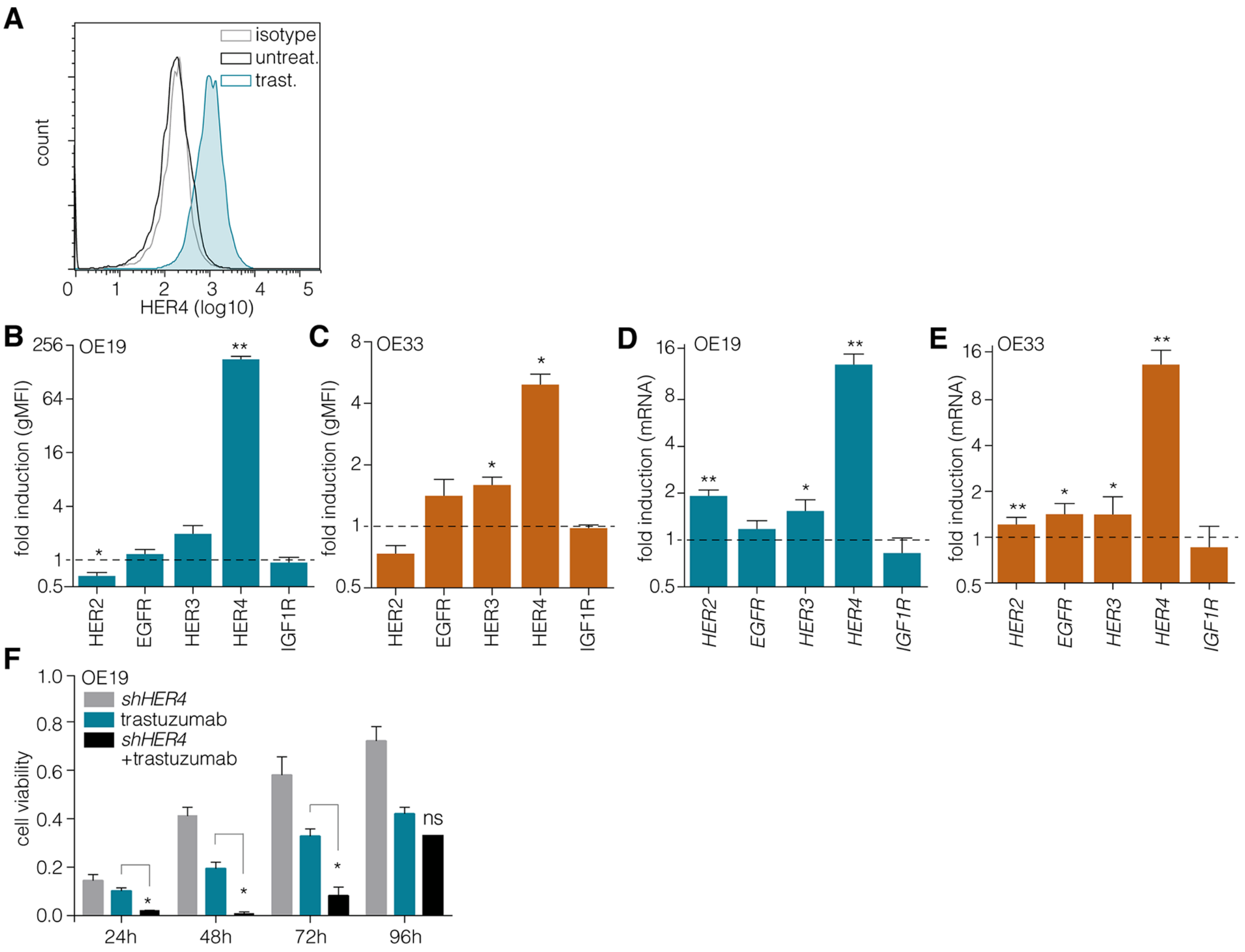

Supplementary Figure S2: HER4 is involved in short-term trastuzumab resistance. A. Representative flow cytometry staining for HER4 on OE19 cells after 48 h PBS control or trastuzumab treatment (1µg/ml). **B, C.** Indicated HER levels on OE19 and OE33 cells upon 1µg/ml trastuzumab treatment (48h) were determined using flow cytometry for indicated proteins. Data were calculated using the gMFI, and values were normalized to the untreated condition (set to 1, not shown in graph). **D, E.** Indicated HER levels were determined by qPCR. Relative expression values were calculated and normalized to GAPDH according to the comparative threshold cycle (Cp) method and normalized to untreated cells. **F.** Cell viability of OE19 cells with doxycycline-induced knockdown of HER4 (shHER4, knockdown efficiency 97%) with or without 1µg/ml trastuzumab were measured using the Cell Titer Blue assay. Data show the mean, relative to control (set to 1). Error bars represent the s.e.m. of three independent experiments. (*P-value <0.05, **P-value <0.01).

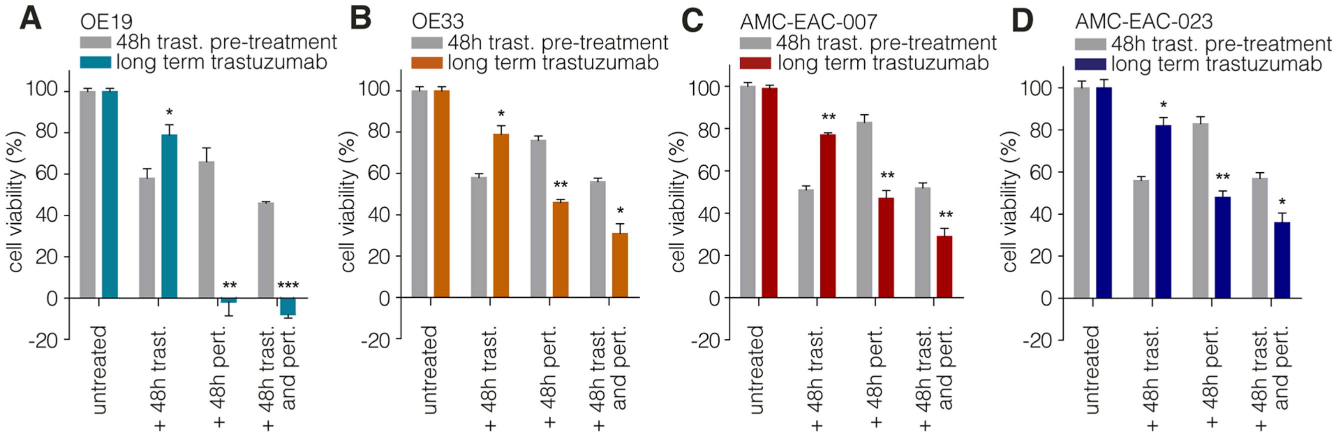

Supplementary Figure S3: Targeting EGFR in trastuzumab resistant cells. The short term treated cells (grey bars) were first treated with trastuzumab for 48h, followed by a 48h incubation with either panitumumab alone or a combination of trastuzumab and panitumumab. As a control , cells were first cultured short-tern (48h) with trastuzumab followed by an additional trastuzumab treatment period of 48h. Trastuzumab indicated on x-axis means that treatment was continued during the experiment along with the other indicated treatments. Cell viability was measured using CTB assay prior to treatment (input) and after treatment. Values are corrected for input and normalized to the control treated condition. Data show the mean ± s.e.m., n≥3. (* = P-value <0.05, ** = P-value <0.01, *** = P-value <0.001).

Supplementary Table S1: Primer sequences for shRNA cloning and PCR

transcript	sequence
shHER4 Fw	CCGGCCGGCCTGTGGCTATTAAGATTCTTCTCGAGAAGAATCTTAATAGCCACAGGTTTTT
shHER4 Rv	AATTAAAAACCTGTGGCTATTAAGATTCTTCTCGAGAAGAATCTTAATAGCCACAGGCCGG
ADAM10 sh1	GCAGGTTCTATCTGTGAGAAA
ADAM10 sh2	GCTGTGCAGATCATTCAGTAT
ADAM17 sh1	CCTGGTTACAACTCATGAATT
ADAM17 sh2	CCTATGTCGATGCTGAACAAA
EGFR Fw	GTGATCCAAGCTGTCCCAAT
EGFR Rv	ACTGGTTGTGGCAGCAGTC
HER3 Fw	TGGGGAACCTTGAGATTGTG
HER3 Rv	GAGGTTGGGCAATGGTAGAG
HER4 Fw	CATGGCCTTCCAACTTGACT
HER4 Rv	TCTGGCAATGATTTTCTGTGG
IGF1R Fw	GTCGAAGAATCGCATCATCA
IGF1R Rv	GCATCCTGCCCATCATACTC
